# The Role of α1-Adrenoceptor Antagonists in the Treatment of Prostate and Other Cancers

**DOI:** 10.3390/ijms17081339

**Published:** 2016-08-16

**Authors:** Mallory Batty, Rachel Pugh, Ilampirai Rathinam, Joshua Simmonds, Edwin Walker, Amanda Forbes, Shailendra Anoopkumar-Dukie, Catherine M. McDermott, Briohny Spencer, David Christie, Russ Chess-Williams

**Affiliations:** 1 School of Pharmacy, Griffith University, Gold Coast, QLD 4222, Australia; mallory.batty@griffithuni.edu.au (M.B.); rachel.pugh@griffithuni.edu.au (R.P.); ilampirai.rathinam@griffithuni.edu.au (I.R.); joshua.simmonds@griffithuni.edu.au (J.S.); edwin.walker@griffithuni.edu.au (E.W.); s.dukie@griffith.edu.au (S.A.-D.); b.spencer@griffith.edu.au (B.S.); David.Christie@genesiscare.com.au (D.C.); 2 Centre for Urology Research, Faculty of Health Sciences and Medicine, Bond University, Robina, QLD 4226, Australia; amanda.forbes@student.bond.edu.au (A.F.); camcderm@bond.edu.au (C.M.M.); 3 Menzies Health Institute Queensland, Griffith University, Gold Coast, QLD 4222, Australia

**Keywords:** α1-adrenoceptor antagonist, prostate cancer, cytotoxicity

## Abstract

This review evaluates the role of α-adrenoceptor antagonists as a potential treatment of prostate cancer (PCa). Cochrane, Google Scholar and Pubmed were accessed to retrieve sixty-two articles for analysis. In vitro studies demonstrate that doxazosin, prazosin and terazosin (quinazoline α-antagonists) induce apoptosis, decrease cell growth, and proliferation in PC-3, LNCaP and DU-145 cell lines. Similarly, the piperazine based naftopidil induced cell cycle arrest and death in LNCaP-E9 cell lines. In contrast, sulphonamide based tamsulosin did not exhibit these effects. In vivo data was consistent with in vitro findings as the quinazoline based α-antagonists prevented angiogenesis and decreased tumour mass in mice models of PCa. Mechanistically the cytotoxic and antitumor effects of the α-antagonists appear largely independent of α 1-blockade. The proposed targets include: VEGF, EGFR, HER2/Neu, caspase 8/3, topoisomerase 1 and other mitochondrial apoptotic inducing factors. These cytotoxic effects could not be evaluated in human studies as prospective trial data is lacking. However, retrospective studies show a decreased incidence of PCa in males exposed to α-antagonists. As human data evaluating the use of α-antagonists as treatments are lacking; well designed, prospective clinical trials are needed to conclusively demonstrate the anticancer properties of quinazoline based α-antagonists in PCa and other cancers.

## 1. Introduction

Prostate cancer is the most commonly diagnosed male cancer in the world [1]. In Australia, prostate cancer account for approximately 30% of all newly diagnosed cancers and is the second most common cause of cancer-specific death in men [2]. Early stage prostate cancer is highly manageable using definitive radical prostatectomy and/or radiotherapy techniques. However, an estimated one-fifth of men will experience disease recurrence following curative treatment modalities [3,4,5] and resort to first-generation androgen deprivation therapies for long-term management of their disease. Unfortunately, progression after androgen deprivation therapy indicates the transition to castrate-resistant prostate cancer (CRPC), which is considered to be both inevitable and incurable. Although there has been significant progress in the CRPC treatment landscape (e.g., enzalutamide, abiraterone, cabazitaxel), there are no currently available therapies which provide a survival benefit greater than twelve months [6,7,8,9,10]. Therefore, there is an urgent need for novel agents to improve the oncological and survival outcomes for these last-resort patients. One such modality may be through the use of α1-adrenoceptor (ADR) antagonists.

Adrenoceptors (also known as adrenergic receptors) are members of the G protein-coupled receptor (GPCR) superfamily, which can be further broken down into α and β subtypes with several homologous isoforms including α-1 (A, B, and D), -2 (A, B, and C), and β-1, 2, and 3 [11]. While all adrenergic receptors play an important role in regulating human tissue homeostasis, the focus of this review will primarily cover α1-ADRs in the human prostate. α1-ADRs are largely found in the stromal region of the human prostate, with few α1-ADR receptors localised in the prostate epithelium. Although, the α1A-ADR isoform (previously identified as α1C) is known to make up approximately 70% of the prostatic α1-ADRs [12], recent evidence suggests that the distribution of α1-ADR isoforms (A, B and D) change with advancing age and are correlated with the subsequent onset of prostatic hyperplasia [13]. Likewise, receptor localisation and expression appears to be altered in prostate cancer tissues. Unlike normal prostate epithelium which expresses few α1-ADRs, prostate cancer epithelia have been reported to express functional α1A-ADR [14,15], as well as increased mRNA levels of α1B and α1D isoforms [16]. It remains unclear whether α1-ADRs have a role in promoting prostate carcinogenesis remains unclear. However, α1-ADRs have been identified to play a role in cellular proliferation in vitro [14,17,18,19] and therefore may be exploited for treatment of neoplasms.

α1-ADR antagonists (referred to here as “α-antagonists”) are commonly used in clinical practice to treat hypertension, and more recently, the urodynamic symptoms associated with benign prostate hyperplasia (BPH). In BPH, α-antagonists block receptor activation to relax the prostatic smooth muscle thereby improving rate of urine flow and other associated lower-urinary tract symptoms (LUTS) [20,21]. There are regional differences in the commonly prescribed α-antagonists for BPH. In the United States, the non-selective doxazosin and terazosin are the most commonly prescribed α1-blockers due to their relatively long half-life [22,23] and clinically significant improvement in BPH-related LUTS. Furthermore, these drugs have been associated with fewer adverse drug-related cardiovascular side effects, compared to prazosin [24]. However, in Australia, the short acting and non-selective prazosin is clinically favored over other α-antagonists primarily due to the rapid mitigation of LUTS. The highly selective tamulosin, also offer significant reduction in BPH-related LUTS symptoms, however, at a cost of ejaculatory dysfunction making this α1-ADR antagonists undesirable for some men [24].

In the late 1990s, monotherapy with α-antagonists was shown to provide long-term clinical benefits that could not be explained solely by acute prostatic relaxation [25,26,27]. In support of these findings, a more recent study uncovered a large proportion of men (70%) experienced continued improvement of BPH-associated LUTS following discontinuation of α-antagonists [28]. Subsequent studies over the next sixteen years have identified that some of these drugs possess novel cytotoxic actions in diseased prostates, including prostate and other cancers. Despite the plethora of original papers investigating the anticancer effects of these drugs, only few systematic reviews since the early 2000s have been carried out to colligate the more recent published findings [29,30,31,32,33]. Therefore, the aim of this systematic literature review is to analyse the current evidence for the use of α-antagonists as potential treatment options for prostate cancer (PCa). Specifically, this review will colligate the anticancer mechanisms of α-antagonists, evaluate the evidence supporting clinical anticancer efficacy of these drugs in PCa, and evaluate the evidence for use of these drugs in other cancers.

## 2. Results

**Figure A1:**
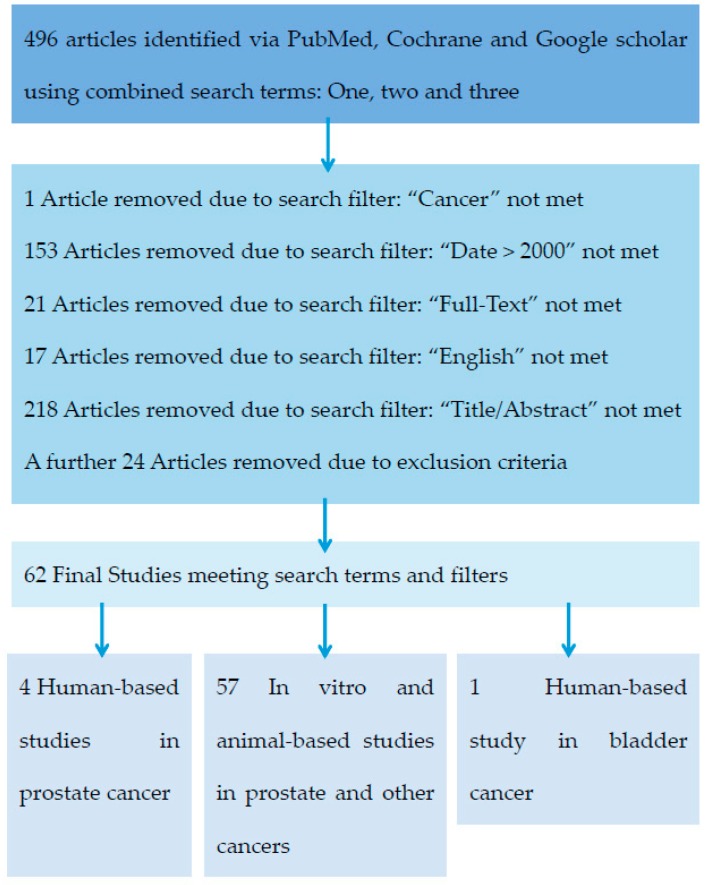
Stepwise process used to determine the final list of studies.

Pubmed, Google Scholar and Cochrane databases were accessed to retrieve articles. The search terms used to find the relevant articles were separated into three categories: terms that describe the α-antagonists, the target tissue and the action of the drugs (Table A1). Four hundred and ninety-six articles were identified using the inclusion criteria by searching three databases: Cochrane, Pubmed and Google Scholar. After exclusion criteria were applied, sixty-two relevant articles were obtained, consisting of fifty-four original manuscripts and eight review articles. (Figure A1). Of the fifty-four research articles identified only four studies examined the role of α-antagonists in PCa development in humans (Table 1). The majority focused on the cytotoxic and anti-tumour activity of α-antagonists in vitro and in animal models (Table 2). These retrospective cohort and observational human studies examined the effects of both quinazoline and non-quinazoline α-antagonists, but show only an overall decreased incidence of PCa. Two additional researchers replicated the search which returned identical results, validating the method used, for robustness.

## 3. Discussion

The overall aim of this literature review was to analyse the current evidence for the clinical use of α-antagonists as a potential treatment modality for PCa. A summary of results from the sixty-two studies identified in this systematic review can be found in Table 2.

### 3.1. In Vitro Evidence

#### 3.1.1. Quinazoline/Piperazine-Dependence

**Figure 1:**
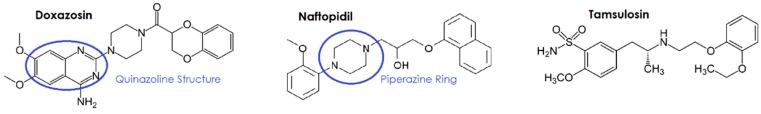
Structural comparison of the α-antagonists doxazosin, naftopidil and tamsulosin.

In vitro studies provide substantial evidence that the quinazoline α-antagonists doxazosin, terazosin and prazosin exhibit cytotoxic activity in the prostate cancer cell lines LNCaP (androgen-dependent), DU145 and PC-3 (castrate-resistant) cell lines [38,39,40,41,42,43,44,45,46,47,48,49,50,51,52,53,84,85,86,87]. The structurally similar piperazine, naftopidil, also produced cytotoxic effects in the androgen-dependent LNCaP and E9 cell lines [88]. However these effects were not seen with the sulphonamide based tamsulosin [88] suggesting that the quinazoline/piperazine ring structure maybe responsible for their cytotoxicity (Figure 1). Furthermore, a number of studies have also investigated the use of doxazosin and naftopidil analogues, which demonstrated similar cytotoxic potential to the parent drug [54,55,89,90].

#### 3.1.2. α1-Adrenoceptor-Independence

The mechanisms for cytotoxicity appear to be independent of α1-blockade [39,40,41] as demonstrated by several studies, through the use of phenoxybenzamine (a non-selective, irreversible α-antagonist). Doxazosin and terazosin were observed to reduce cell viability and induce apoptosis in the presence of phenoxybenzamine [32,38,42,43]. This independent action is supported by two studies that proposed involvement of the 5HT receptor [44,89] which resulted in reduced cell viability and apoptosis in PCa.

#### 3.1.3. Cell Death Mechanisms

**Figure 2:**
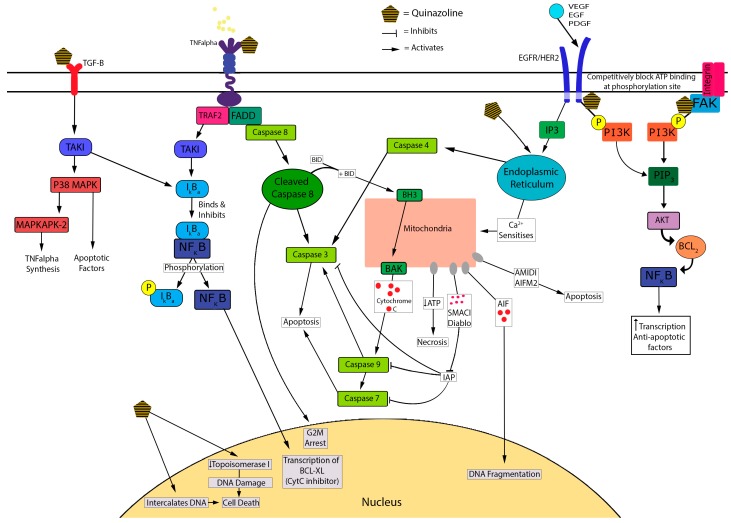
Proposed cytotoxic mechanisms underlying quinazoline-based α-antagonists.

There are several potential mechanisms accounting for the cytotoxic actions of the quinazoline/piperazosine α-antagonists, including apoptosis, decreased cell proliferation and decreased angiogenesis which are crucial mediators of quinazoline-induced cytotoxicity in PCa cell lines [38]. An illustrative summary of the mechanisms contributing to α-antagonist-induced cytotoxicity is shown in Figure 2.

Early work by Kyprianou, N. et al. (2000) [32] showed that doxazosin (15 mM) and terazosin (15 mM) induce apoptosis in a dose dependent manner in PC-3 cell lines using the TUNEL assay [38,42]. As well as inducing apoptosis, doxazosin and terazosin were shown to inhibit cell adhesion to the extracellular matrix by inducing anoikis. Both agents induced apoptosis in prostate epithelial and smooth muscle cells at dose ranges used for the treatment of BPH [38]. Similarly Garrison, J. et al. (2006) [45] proposed that apoptosis was an important mediator of doxazosin-induced cytotoxicity (at 25 mM/L) in both malignant and benign prostate epithelial cells (PC-3 and BPH-1 cell lines). These authors suggested that this occurs through increased caspase 8 activation via formation of the death-inducing signalling complex (DISC) [32,45]. Caspase 8 mediates cell cycle arrest at the G2-M phase [46], and activates both cleaved caspase 3 and tBid at the BAX/Bak receptor [32,56]. This results in the release of mitochondrial stress related pro-apoptotic inducing factors including: cytochrome C, Smac/diablo, AMID and AIF [29,35,47,91]. More recently, Forbes, A. et al. (2016) [47] found that in PCa cells, the activation of caspase 3 was similar for prazosin and doxazosin, and suggested superior activity to terazosin, silodosin and alfuzosin. Prazosin (30 mM) treatment resulted in a six-fold increase in caspase 3 activation in LNCaP versus a two-fold increase in PC-3 cells suggesting androgen-dependent prostate cencer (ADPC) cells have greater sensitivity to these effects [47]. Cleaved caspase 3 is used as a marker for apoptosis [32] and is activated via DISC through FADD recruitment [32,45]. Some studies support Forbes’ finding of a dose-dependent increase in caspase 3 activation and consequent apoptosis when treated with quinazolines [32,47,48]. A decrease in HIF-1 (a mediator of resistance) was also shown in LNCaP cells post quinazoline exposure [47].

Additionally, α-antagonists exhibit cytotoxicity via cell-cycle arrest. Naftopidil induced G1 cell-cycle arrest in PCa cells in vitro, as did silodosin, but to a lesser extent [88]. Similarly, prazosin and doxazosin caused an increase in DNA strand breakage leading to subsequent G2 cell-cycle arrest and apoptosis, possibly through the inactivation of CDK1 [46,57]. Ho, C. et al. (2015) [58] showed that the reversible non-selective α-antagonist, phentolamine (an imidazoline), caused cell-cycle arrest in CRPC cells by inducing microtubule assembly, leading to mitotic arrest of the cell-cycle and mitochondrial damage. This inhibition of mitosis is a similar chemotherapeutic mechanism to taxanes [59]. It was suggested that disruption of the cell-cycle by quinazolines can be explained by competitive inhibition of the ATP attachment of tyrosine kinase and inhibiting phosphorylation of PI3K from the following receptors: HER2/Neu, EGF, and VEGF [32,60]. These receptors are well-identified targets of current chemotherapeutic agents, such as bevacizumab which targets VEGF.

Another proposed mechanism underlying the cytotoxic actions of α-antagonists is disruption of DNA integrity. Desiniotis, A. et al. (2011) [32] suggested that quinazolines derivatives cause DNA intercalation, similar to anthracycline chemotherapeutics. DNA fragmentation was also observed in studies that tested doxazosin (25 mM) [32,49]. Doxazosin is proposed to inhibit topoisomerase 1, inducing DNA damage and resulting in synergistic cytotoxic activity with etoposide and adriamycin [86]. Furthermore, apoptosis and cell-cycle arrest lead to decreased cell growth and proliferation of PCa cells. This leads to decreased cell survival, migration and adhesion resulting in anoikis [50,61,90].

In vitro evidence also suggests that quinazolines have the potential to disrupt key mediators of angiogenesis. Quinazolines downregulate VEGF, resulting in reduced repression of TGF-β receptor [32,51,60]. TGF-β is responsible for the transcription of various apoptosis factors as well as increasing IκBα; the inhibitor of NF-κB [32,60]. Forbes, A. et al. (2016) [51] noted an increase in stress related factors such as p38α and MAPKs in PCa cells treated with α-antagonists [47], which is suggestive of TGF-β activation. The α-antagonist-mediated disruption and down-regulation of VEGF results in decreased angiogenesis by increasing apoptosis and anoikis [32,52,61]. The inhibition of this signalling pathway blocks Bcl-2, an anoikis inhibiting factor that is identified in CRPC and is a mediator for cell immunity via bypass pathway mutations [32,52]. Targeting this factor improves selectivity and may improve treatment outcomes in CRPC. This was observed in prostate cells, where treatment with doxazosin resulted in inhibition of VEGF-induced angiogenesis, reduced cell migration and increased cell death due to anoikis [53,61], possibly via EphA2 agonist activity [62].

### 3.2. In Vivo Evidence

Consistent with in vitro studies, the ability of quinazoline α-antagonists to reduce tumour growth and potentially decrease angiogenesis is also observed in mice models of PCa. PCa xenografts in mice showed that tumour mass was significantly reduced when treated with quinazoline compounds prazosin and doxazosin compared to untreated controls, possibly through the induction of apoptosis [38,42,46,57,62]. Terazosin treatment in nude mice significantly reduced VEGF induced angiogenesis. This effect was also seen in prostate tumour mice models [39] suggesting that terazosin has very potent anti-angiogenic effects, reducing tumour volume over time. Anti-proliferative effects of doxazosin were also observed in Wistar rats treated with doxazosin and finasteride [63]. Interestingly, doxazosin has recently been identified as a novel EphA2 agonist [47,62], which triggers PCa cytotoxicity via cell rounding and detachment in vitro and this mechanism may translate to animal models [62]. In line with previous in vivo studies, doxaozisn was previously found to reduce tumour metastasis and improve survival of PC-3 xenograft nude mice. These anti-tumour effects were proposed to occur, to some extent, by EphA2-mediated cell detachment, inhibition of tumour cell migration [62], and indirect activation of apoptosis.

### 3.3. Clinical Evidence

To determine if the cytotoxic and anti-tumour effects observed in vitro and in mice models translate into a potential therapeutic application in human patients, we examined their effects in patients taking them long term (ranging from 3 to 11 months or longer) [34,36]. However, to date there are only four retrospective human studies that have investigated the benefit of quinazoline based α-antagonists in patients after original treatment ended (Table 1). Interestingly, both quinazoline and non-quinazoline α-antagonists appear to decrease the incidence of PCa at doses indicated for the symptomatic relief of LUTs [35,36,37,64]. It is therefore difficult to evaluate their potential for treating PCa.

### 3.4. Anticancer Effects of α-Antagonists in Other Cancers

Lastly, we examined the potential of cytotoxic and antitumor actions of α-antagonists in other cancers (See Table 2 and Supplementary Material Figure S1 for detailed review). Consistent with in vitro and in vivo animal findings in PCa, classical α-antagonists and their analogues appear to have broad activity, exhibiting cytotoxicity in other cancer cell lines including urogenital [44,65,66,67,68,69,70,71,72,73,74], gastrointestinal [73,74,75,76,77], lung [74,78], blood [79], brain [80,81] and thyroid [82]. Importantly, the cytotoxic and anti-proliferative effects are supported by several in vivo mice studies [48,66], suggesting ubiquitous anticancer actions of these drugs. In support of in vitro findings a retrospective study in 24 patients with bladder cancer, 15 of which had been treated with terazosin over a 3–6 month period, had a reduction in incidence, tissue MVD and increase in apoptotic index [83]. The only trials with doses that are of clinical relevance are with bladder, pituitary and ovarian cancer, all of which are within the standard dosage ranges of the respective medications [48,50,66]. The proposed cytotoxic mechanisms of α-antagonists in other cancers differ from those identified in PCa, suggesting the magnitude of their anticancer effects may vary between cancer types. It is difficult to draw sound conclusions of the efficacy of α-antagonists in other cancers from this data.

## 4. Conclusions

PCa is the most commonly diagnosed cancer in Australia with substantial mortality associated with the castrate resistant form. Current treatments are significantly limited by the development of resistance, as well as severe toxicity. Therefore, there is an urgent need to identify alternate or adjunct treatment options. Given their key role in managing the LUTs associated with PCa and its treatments, as well as in vitro cytotoxicity against PCa cell lines, α-antagonists may offer such a treatment option. However, apart from a series of excellent in vitro studies and limited animal studies, the potential of α-antagonists as a treatment option in human patients remains unclear. Therefore, the purpose of this literary review was to analyse the current evidence for the use of α-antagonists in the treatment of prostate and other cancers, and elucidate mechanisms responsible for their cytotoxic effects.

Several elegant in vitro studies demonstrate that quinazoline based α-antagonists (doxazosin, terazosin and prazosin) are cytotoxic to PCa cell lines by inducing apoptosis, inhibiting cell proliferation and angiogenesis. Similarly, these effects were also observed with the piperazine based agent naftopidil. In contrast tamsulosin, a sulphonamide based compound did not exhibit cytotoxic activity, suggesting that structural specificity is important in eliciting cytotoxic action. In vitro studies also suggest that the quinazoline α-antagonists may also target angiogenesis by disrupting VEGF. Furthermore, several studies also suggest that the cytotoxic actions are not limited to PCa cell lines as α-antagonists were also shown to induce cell death in some of the following cell lines: Bladder HT1376, Ovarian SKOV-3, Renal Carcinoma ACHN and Caki-2. However, more robust trials with standardised methodologies are required to strengthen the evidence of α-antagonists as chemotherapeutic agents in cancers other than prostate.

The in vitro findings are reflected in mice models of PCa, with many studies showing that tumour growth and angiogenesis is significantly decreased when animals are treated with quinazoline or piperazine agents. However, evidence for the potential of the quinazoline and piperazine α-antagonists to treat PCa in human patients is lacking. We identified only four studies looking at the risk of developing PCa in human patients using α-antagonists. These retrospective and observational cohort studies did not examine the potential of these agents to treat PCa. Instead, they showed a decreased incidence of PCa in long term users of α-antagonists.

Therefore, while the in vitro and animal studies clearly demonstrate the potential role of quinazoline and piperazine based α-antagonists in the treatment of prostate and other cancers, well designed, prospective clinical trials in humans are required to ultimately evaluate their efficacy as either a primary treatment option or as an adjunct. It is difficult to draw sound conclusions of the efficacy of α-antagonists in other cancers from the data analysed in this review. More robust trials with standardised methodologies are required to strengthen the evidence of α-antagonists as chemotherapeutic agents in cancers other than prostate. We hope the findings from this literature review will stimulate further research to potentially place α-antagonists as possible treatment options for PCa in the future.

## Figures and Tables

**Table 1:** Clinical-based studies investigating the effect of α1-antagonists on prostate cancer (PCa).

Author	Title	Drug	Results	Study Type
Keledjian, K. et al. [34]	Reduction of human prostate tumor vascularity by the α1-adrenoceptor antagonist terazosin	Terazosin	Increased apoptotic index in prostate carcinoma after terazosin treatment. Reduction in prostate tumour vascularity in terazosin-treated BPH patients. Patients were treated for 6–11 months	Retrospective Cohort study
Harris, A. et al. [35]	Effect of α1-adrenoceptor antagonist exposure on prostate cancer incidence: an observational cohort study	Doxazosin & Terazosin	4070 men were treated with α-antagonists for Benign prostatic hyperplasia or hypertension or HTN. The incidence of PCa among treated men vs. untreated men was 1.65% and 2.41% respectively. Data showed 7.6 fewer cases developed per 1000 exposed men	Observational Cohort study
Yamada, D. et al. [36]	Reduction of prostate cancer incidence by naftopidil, an α1-adrenoceptor antagonist and transforming growth factor-β signalling inhibitor	Naftopidil & Tamsulosin	PCa incidence was significantly lower in men treated with naftopidil for ≥3 months compared to men treated with tamsulosin. (*p* = 0.035)	Retrospective Cohort study
Bilbro, J. et al. [37]	Therapeutic value of quinazoline-based compounds in prostate cancer	Doxazosin, terazosin and other quinazolines	Patients treated with α_1_-antagonists: doxazosin and terazosin, at the Markey Cancer centre had reduced risk of developing PCa	Retrospective Cohort study

**Table 2:** Summary of identified studies investigating the anticancer effect of α-antagonists.

Ref.	Author	Title	Study Type	Cancer Type	Drugs	Findings (Original Studies)
[29]	Nishizaki, T. et al.	1-[2-(2-Methoxyphenylamino) ethylamino]-3-(naphthalene-1-yloxy) propan-2-ol may be a promising anticancer drug	Review	NA	NA	
[30]	Kyprianou, N. et al.	Apoptosis induction by doxazosin and other quinazoline α1-adrenoceptor antagonists: a new mechanism for cancer treatment?	Review	NA	NA	
[31]	Patane, S. et al.	Insights into cardio-oncology: Polypharmacology of quinazoline-based α1-adrenoceptor antagonists	Review	NA	NA	
[32]	Desiniotis, A. et al.	Advances in the design and synthesis of prazosin derivatives over the last ten years	Review	NA	NA	
[33]	Tahmatzopoulos, A. et al.	The role of α-blockers in the management of prostate cancer	Review	NA	NA	
[34]	Kyprianou, N. et al.	Suppression of human prostate cancer cell growth by α1-adrenoceptor antagonists Doxazosin and terazosin via induction of apoptosis	In vitro, in vivo (mice)	Prostate Cancer (PC-3 and DU145)	Doxazosin, terazosin, tamsulosin, phenoxy-benzamine	Doxazosin and terazosin induced apoptosis in prostate epithelial and smooth muscle cells in patients with BPH, without affecting rate of cell proliferation in PCa cells. This effect could not be prevented by irreversible inhibition of α1-adrenoceptors (phenoxybenzamine), indicating an in vitro toxicitity occurs via an α-receptor independent mechanism. Doxazosin administration (at tolerated pharmacologically relevant doses) in SCID mice resulted in a significant inhibition of PC-3 tumour growth, presumably via induction of apoptosis.
[35]	Pan, S. et al.	Identification of apoptotic and antiangiogenic activities of terazosin in human prostate cancer and endothelial cells	In vitro, in vivo (mice)	PCa PC-3 & endothelial HUVEC cells	Terazosin	It was found that terazosin induced apoptosis in PC-3 and human benign prostatic cells (IC_50_ > 100 µM), and possessed potent anti-angiogenic effect in endothelial cells compared to PCa cells. Terazosin (IC50 of 7.9 µM) significantly inhibited VEGF-induced angiogenesis and endothelial tube formation in nude mice, demonstrating that terazosin had a more potent anti-angiogenic than cytotoxic effects. Terazosin also effectively inhibited vascular endothelial growth factor induced proliferation and tube formation in cultured human umbilical vein endothelial cells (IC50 9.9 and 6.8 µM, respectively). Doxazosin, but not tamsulosin, mimicked these effects and the anti-tumour effects of these drugs were determined to occur independent of α1-adrenoceptor antagonizing activity.
[36]	Walden, P. et al.	Induction of anoikis by Doxazosin in prostate cancer cells is associated with activation of caspase-3 and a reduction of focal adhesion kinase	In vitro	PCa (PC-3 and LNCaP)	Doxazosin	Doxazosin induced changes in morphology consistent with anoikis in both benign and cancerous prostatic cells (rounding up of cells, DNA-degradation in the nucleus, cell shrinkage, the appearance of vacuoles, and cell detachment from the tissue culture plate) and increased caspase-3 activity. The increase of caspase-3 activity by doxazosin promotes anoikis and, subsequently, apoptosis of cancer cells. Treatment of PC-3 cells with doxazosin significantly reduced the protein levels of anti-anoikis kinase, FAK, but did not significantly affect the levels of ILK. Norepinephrine had no effect on doxazosin-induced cell morphology or caspase-3 activity, indicating that the apoptotic/anoikis effects of doxazosin result from mechanism that is a_1_-adrenoceptor independent.
[37]	Benning, C. et al.	Quinazoline-derived α1-adrenoceptor antagonists induce prostate cancer cell apoptosis via an α1-adrenoceptor-independent action	In vitro	Prostate cancer cells	Doxazosin, terazosin	Transfection-mediated overexpression of α1-adrenoreceptors in human prostate cancer cells, DU-145 (AR-independent, and reportedly lack of adrenoceptors), did not alter the ability of prostate cancer cells to undergo apoptosis in response to quinazolines. These findings indicate that apoptotic activity of quinazoline-based α1 adrenoceptor antagonists (doxazosin and terazosin) in prostate cancer cells is independent of α1-adrenoceptor antagonism.
[38]	Kyprianou, N.	Doxazosin and terazosin suppress prostate growth by inducing apoptosis: clinical significance	Review, in vitro, in vivo (mice)	PC-3, DU-145 and SMC-1	Doxazosin, terazosin	Doxazosin and terazosin significantly reduced the viability of PC-3 and LNCaP cells by inducing caspase-3 mediated apoptosis in a dose dependent manner, however only doxazosin induced significant death of PCa cells. Doxazosin (and not terazosin) significantly affect the rate of proliferation of PCa cells. Irreversible inhibition with phenoxybenzamine did not abolish the apoptotic effect of doxazosin or terazosin against PCa or SMC cells, indicating the cytotoxic effects occurred via an α1-independent mechanism. Oral treatment with doxazosin resulted in significant decrease in tumour volume of PCa xenografts compared to controls, presumably via induction of apoptosis.
[39]	Arencibia, J. et al.	Doxazosin induces apoptosis in LNCaP prostate cancer cell line through DNA binding and DNA-dependent protein kinase down-regulation	In vitro	LNCaP	Doxazosin	Doxazosin induced dose-dependent LNCaP cytotoxicity and apoptosis, which could not be prevented by phenoxybenzamine, indicating an α1-adrenoceptor independent cytotoxicity. Microarray analysis following doxazosin treatment (8–24 h, 20 µM) identified 70–92 deregulated genes, including several involved in cell-cycle control and drug response, and a few related to other cellular processes such as apoptosis or angiogenesis. An inverse correlation was observed with doxazosin concentration and topoisomers, suggesting that topoisomerase I is inhibited by the binding of doxazosin to DNA. Thus, doxazosin could cause DNA damage, resulting in apoptotic cell death.
[40]	Siddiqui, E. et al.	Growth inhibitory effect of Doxazosin on prostate and bladder cancer cells. Is the serotonin receptor pathway involved?	In vitro	PCa PC-3, bladder cancer HT1376	Doxazosin	Doxazosin was found to significantly reduce PCa PC-3 and bladder cancer HT1376 cell growth, which was partially prevented through pre-treatment with 5HT or 5-HT1B. These findings may be related to the structural similarity between subtype 1 serotonin and adrenergic receptors, and the authors suggests that doxazosin displaces 5-HT from 5-HT receptors.
[41]	Garrison, J. et al.	Doxazosin induces apoptosis of benign and malignant prostate cells via a death receptor-mediated pathway	In vitro	PC-3 and BPH1	Doxazosin	Doxazosin (24 h) causes a dose dependent loss of cell viability and induces apoptosis in PC-3 and BPH1 cells 24 h after treatment. After a short doxazosin treatment (6 h), several genes that play a critical role in apoptosis were upregulated in PC-3 cells. In particular, doxazosin was found to upregulate Bax mRNA transcription and induced caspase-8 mediated apoptosis.
[42]	Lin, S. et al.	Prazosin displays anticancer activity against human prostate cancers: targeting DNA and cell cycle	In vitro, in vivo (mice)	Prostate Cancer	Prazosin	Prazosin exhibited anti-proliferative activity superior to that of other α-blockers. It induced G2 checkpoint arrest and subsequent apoptosis. In PC-3 cells, prazosin increase in DNA strand breakage leading to Cdk1 inactivation and subsequent cell cycle arrest. In mice, prazosin significantly reduced tumour mass in PC-3 derived cancer xenografts.
[43]	Forbes, A. et al.	Relative cytotoxic potencies and cell death mechanisms of α-adrenoceptor antagonists in prostate cancer cell lines	In vitro	PCa PC-3, LNCaP	Prazosin Doxazosin, terazosin, silodosin, alfuzosin, tamsulosin	The relative potency order was prazosin = doxazosin > terazosin = silodosin = alfuzosin> tamsulosin on both cell types, but LNCaP cells were significantly more sensitive to these effects that PC-3 cells. Prazosin and doxazosin increased levels of apoptosis and autophagy in both cell lines. However, autophagy was found to play a paradoxical role by contributing to survival of LNCaP and cytotoxicity of PC-3 cells. Treatment with prazosin (30 µM) altered the expression of several cell stress-related proteins: elevating phospho-p38α and reducing S6 kinase in both cell lines. The expression of some proteins were differentially affected in PC-3 and LNCaP cell; Akt and p27 increasing and HIF-1α decreasing in LNCaP cells but not PC-3, while ADAMTS1 was increased in PC-3 cells only. Phosphorylation of EphA2 was also reported to play a role in doxazosin, but not prazosin, induced PC-3 cytotoxicity.
[44]	Fernando, M. et al.	α1-Adrenergic receptor antagonists: novel therapy for pituitary adenomas	In vitro, in vivo (mice)	Pituitary tumour	Doxazosin	Treatment with Doxazosin results in reduced phosphorylation and down-regulation of NF-κB. Decreased phosphorylated retinoblastoma and PCNA expression, which resulted in cell cycle arrest at G0-G1. Doxazosin treatment also increased cleaved caspase 3. In mice, the tumour mass was lower in the doxazosin treated group. In contrast to current literature, this study suggested that the cytotoxic activity of quinazoline-antagonists was greater in cells that express α1a and 1b. In addition to apoptosis, doxazosin treatment appeared to reduce the circulating ACTH level and therefore may be useful for symptomatic relief.
[45]	Youm, Y. et al.	Doxazosin-induced clusterin expression and apoptosis in prostate cancer cells	In vitro	PCa PC-3	Doxazosin	Doxazosin-induced DNA fragmentation after 24 h treatment, and was statistically significant after 48 h treatment of PC-3 cells. Clusterin expression in PC-3 cells was 3-fold higher in doxazosin treated cells (9 h) compared to untreated controls, and was maintained over 48 h. These findings were found to be consistent with doxazosin-induced apoptosis. Immunocytochemistry analysis (after 9 and 12 h treatment) demonstrated the presence of clusterin in 7% and 18% of total cells respectively. At 24 h treatment, clusterin protein was mainly observed in the cytoplasm and rarely in the nuclei of healthy cells.
[46]	Tahmatzopoulos, A. et al.	Maspin sensitizes prostate cancer cells to Doxazosin-induced apoptosis	In vitro	PCa DU-145	Doxazosin	Maspin (tumour suppressor protein) was shown to increase sensitivity of PCa DU-145 cells to doxazosin, by affect the migration and attachment of malignant prostate cells to the ECM. Also caused mammary MDA-MB-435 cells to undergo apoptosis via increased Bax and caspase-3 activation.
[47]	Partin, J. et al.	Quinazoline-based α1-adrenoceptor antagonists induce prostate cancer cell apoptosis via TGF-β signalling and I κB α induction	In vitro	PCa PC-3	Doxazosin, tamsulosin	Doxazosin, but not tamsulosin, was found to induce PC-3 apoptosis by enhancing TGF-β1 signalling, and subsequently, downstream 1κBα.
[48]	Keledjian, K. et al.	Anoikis induction by quinazoline based α1-adrenoceptor antagonists in prostate cancer cells: antagonistic effect of bcl-2	In vitro	PCa PC-3	Doxazosin, terazosin, tamsulosin (at therapeutic doses)	Treatment of PC-3 cells with doxazosin or terazosin, but not tamsulosin, resulted in significant down regulation of VEGF. Doxazosin also promoted anoikis. However, these effect was reduced in PC-3s that over-expressed Bcl-2 (an anoikis inhibiting factor). In these experimental conditions, these drugs did not have any effect on HIF1-α expression.
[49]	Liao, C. et al.	Anti-angiogenic effects and mechanism of prazosin	In vitro	PCa and HUVEC	Prazosin	Prazosin induced apoptosis in PCa and normal HUVEC cells via different mechanisms, suggesting that prazosin-mediated anti-angiogenic activity and differential modulation of apoptotic pathways are cell-type specific.
[50]	Kim, S. et al.	Dual silencing of Hsp27 and c-FLIP enhances doxazosin-induced apoptosis in PC-3 prostate cancer cells	In vitro	PCa PC-3	Doxazosin	Apoptotic indices increased in a dose-dependent manner when doxazosin was added. In basal conditions (+Hsp27/+c-FLIP), doxazosin (25 µM) induced apoptosis in 52% of cells. In −Hsp27/+c-FLIP cells, apoptotic activity increased to 68% of PC-3 cells. In the opposite case (+Hsp27/−c-FLIP) the apoptotic index was 78%. Even greater number of apoptotic cells were observed (92%) when both Hsp27 and c-Flip were silence. These findings indicate that Hsp27 and c-FLIP play a protective role against doxazosin induced cytotoxicity of PC-3 cells.
[51]	Lee, S. et al.	Expression of heat shock protein 27 in prostate cancer cell lines according to the extent of malignancy and doxazosin treatment	In vitro	PCa LNCaP, PC-3	Doxazosin	RT-PCR studies identified Hsp27 expression to be related to PCa malignancy potential in vitro (e.g., Hsp27 > in PC-3 than LNCaP cells), and was dose-dependently enhanced in some cell lines following doxazosin treatment. Apoptotic cell death triggered by HSP27 siRNA is greater in the androgen receptor-negative cell line PC-3 than in the androgen receptor-positive cell line LNCaP.
[52]	Cal, C. et al.	Doxazosin: a new cytotoxic agent for prostate cancer?	In vitro	PCa DU145, PC-3	Doxazosin adriamycin, etoposide, paclitaxel.	DU-145 and PC3 were sensitive to doxazosin-mediated cytotoxicity, which occurred in a dose- and time-dependent fashion. The combination of doxazosin and adriamycin or etoposide resulted in significant dose-dependent cytotoxic synergism. In contrast, the combination of doxazosin and paclitaxel resulted in antagonistic activity, which was enhanced with increasing concentrations of the drugs.
[53]	Chang, K. et al.	Combined effects of terazosin and genistein on a metastatic, hormone-independent human prostate cancer cell line	In vitro	PCa DU-145	Terazosin, genistein	Terazosin or genistein alone inhibited cell growth in a dose-dependent manner genistein (5 µ/mL) being more effective than terazosin (1 µ/mL—nontoxic dose). Combination treatment significantly increased apoptosis in cells compared to genistein alone. The synergistic effects of these drugs had a greater inhibitory effect the pro-survival Bcl-X_L_ protein, compared to either drug along. Genistein and the combination also were reported to have an effect on angiogenesis-related proteins, causing a significant decrease in VEGF_165_ mRNA and VEGF_121_ mRNA levels.
[54]	Harris, A. et al.	Effect of α1-adrenoceptor antagonist exposure on prostate cancer incidence: an observational cohort study	Observational cohort	PCa	Doxazosin and Terazosin	Incidence of PCa in men exposed to quinazoline-based α-blockers (for BPH or hypertension) was 1.65% whereas in unexposed men, incidence was 2.41%. This indicates men who were exposed to quinazolines were 1.46 times lower relative risk of developing PCa, compared to unexposed men. However, there was no association between quinazoline exposure and overall patient survival.
[55]	Liu, C. et al.	Piperazine-designed α1A/α1D-adrenoceptor blocker KMUP-1 and Doxazosin provide down-regulation of androgen receptor and PSA in prostatic LNCaP cells growth and specifically in xenografts	In vitro, in vivo (mice)	PCa: LNCaP, PC-3 and DU-145	Doxazosin and KMUP-1	KMUP-1 and Doxazosin both inhibit LNCaP cell growth and downregulate expression of AR and PSA. KMUP-1 is a Xanthine derivative PDE inhibitor with α-blocking features. It also has a piperazine moiety very similar to that seen in Doxazosin, naftopidil which is reported to lead to its activity. KMUP-1 significantly inhibited LNCaP cell growth and induced apoptosis in time and dose-dependent manner. KMUP-1 and doxazosin further inhibited the expression of AR and PSA. Treatment of LNCaP cells with KMUP-1 resulted in cell cycle arrest and apoptotic activities, increasing p21 and p27 and decreasing expressions of cyclin D1, cyclin E, cyclin dependent kinase (CDK) 4, CDK2 and CDK6. Moreover, KMUP-1 activated p53, cleaved poly (ADP-ribose) polymerase and caspase-3, but reduced the expression of Bcl-2. Regular administration of KMUP-1 suppressed the LNCaP xenograft tumour growth in nude mice. These evidences indicate that KMUP-1 and doxazosin inhibit LNCaP cell growth and downregulate expression of AR and PSA. KMUP-1 might be used as a chemoprevention agent for preventing the development of prostate cancer without cardiovascular adverse effect of doxazosin.
[56]	Ho, C. et al.	Repurposing of phentolamine as a potential anticancer agent against human castration-resistant prostate cancer: A central role on microtubule stabilization and mitochondrial apoptosis pathway	In vitro	PCa DU145, PC-3	Phentolamine, paclitaxel	Phentolamine induced anti-proliferative effects in PC-3 and Du-145, two CRPC cell lines and p-glycoprotein overexpressing cells. This effect was not significantly reduced in paclitaxel resistant cells. Phentolamine induced mitotic arrest of the cell cycle and formation of hyperdiploid cells, followed by an increase of apoptosis. Mitotic arrest was confirmed by cyclin B1 up regulation, CDK1 activation and a dramatic increase of mitotic protein phosphorylation. In vitro cellular identification demonstrated that phentolamine, similar to paclitaxel, induced tubulin polymerization and formation of multiple nuclei. The Data suggests that phentolamine is a potential anti-cancer agent. It induces microtubule assembly, leading to mitotic arrest of the cell cycle, which ‘in turn’ induces subsequent mitochondrial damage, and activation of related apoptotic signalling pathways in CRPC cells. Furthermore, the combination between phentolamine and paclitaxel induces a synergistic apoptotic cell death. Phentolamine has a simple chemical structure and is not P-gp substrate. Optimization of phentolamine structure may also be a potential approach for further development.
[57]	Anglin, I. et al.	Induction of prostate apoptosis by α1-adrenoceptor antagonists: mechanistic significance of the quinazoline component	Review			
[58]	Keledjian, K. et al.	Doxazosin inhibits human vascular endothelial cell adhesion, migration, and invasion	In vitro	HUVEC, endothelial cells	Doxazosin	Doxazosin results in a dose-dependent loss of cell viability after 24 h of treatment. At concentrations as low as 1 mM, 10% loss of cell viability is observed and at 15 mM there is more than 30% cell death. There is also significant increase in the number of apoptotic cells within 24 h of exposure to doxazosin and a further increase after 48 h. Increased protein expression of pro-caspase-3 was observed after 6 and 12 h of doxazosin treatment. Doxazosin markedly suppresses VEGF—mediated endothelial cell adhesion to fibronectin. HUVEC cells were wounded and 24 h post-wounding, doxazosin treatment (15 mM) resulted in a dramatic decrease in HUVEC cell migration in the absence or presence of exogenous VEGF compared to control. Thus doxazosin can cause suppression of VEGF-mediated cell migration. FGF-2, a potent angiogenic factor, results in significant stimulation of HUVEC angiogenic response that was suppressed by doxazosin treatment. TGF-b had no significant impact on HUVEC-tube formation. Doxazosin treatment for 24 h resulted in a significant downregulation of VEGF mRNA.
[59]	Petty et al.	A small moleculre agonist of EphA2 receptor tyrosine kinase inhibits tumor cell migration in vitro and prostate cancer in vivo	In vitro, in vivo (mice)	PC-3	Doxazosin	Doxazosin induced cell rounding and detachment via agonistic actions on EphA2 in vito. Animal studies found that doxazosin reduced number of tumour metastasis and increased survival in PC-3 xenograft nude mice
[60]	Justulin, L. et al.	Combined effect of the finasteride and doxazosin on rat ventral prostate morphology and physiology	In vivo	PCa	Doxazosin and finasteride	Wistar rats were treated with finasteride and doxazosin and the ventral prostate was excised at day 3 and day 30. The combination induced a transient increase in testosterone plasma concentration and a permanent reduction in DHT. The ventral prostate and epithelial cell proliferation were reduced and the collagen fibre volume fraction and apoptosis of the epithelial cell were increased. Transcription of MMP-2, TIMPs-1 and -2 mRNA was decreased after 30 days of treatment.
[61]	Keledjian, K. et al.	Reduction of human prostate tumor vascularity by the α1-adrenoceptor antagonist terazosin	In vitro, retro-spective	PCa	Terazosin	A significant induction of apoptosis was observed among the cancerous prostatic epithelial cells in the terazosin-treated, as compared to the untreated prostate cancer specimens, while there was no significant change in the proliferative index of the same tumour cell populations after treatment. Furthermore, terazosin resulted in a significant decrease in prostate tissue MVD compared with the untreated group, which correlated with the increased apoptotic index of the cancerous areas. Tissue PSA expression in the prostatic tumour was also markedly reduced after terazosin treatment, while no significant changes in VEGF expression were detected. These findings provide the first evidence that terazosin; a quinazoline-based α-blocker decreases prostate tumour vascularity. Our study has significant clinical implications in identifying selected α-adrenoceptor antagonists as potential anti-tumour agents with apoptotic and anti-angiogenic effects in the human prostate that can be exploited for the treatment of advanced prostate cancer.
[62]	Yamada, D. et al.	Reduction of prostate cancer incidence by naftopidil, an α1-adrenoceptor antagonist and transforming growth factor-β signaling inhibitor	In vitro, retro-spective	PCa	Naftopidil and tamsulosin	Prostate cancer incidence was significantly lower in men who received naftopidil for 3 months or longer compared with tamsulosin (*p* = 0.035). Immunohistochemically positivity for Bcl2, a marker for resistance to apoptosis, was less frequently detected in prostate cancer cells of men who received naftopidil compared with tamsulosin. Naftopidil induced apoptosis and blocked Smad2 phosphorylation activated by transforming growth factor-B in cell lines.
[63]	Tahmat-zopoulos, A.	Apoptotic impact of α1-blockers on prostate cancer growth: a myth or an inviting reality?	Review, in vitro, retrospective	PCa	Terazosin, doxazosin and tamsulosin	Description of α-antagonist induced anoikis and angiogenesis. Discusses retrospective study of patients using terazosin, and marked increase in tumour vascularity on autopsy.
[64]	Bilbro, J. et al.	Therapeutic value of quinazoline-based compounds in prostate cancer	Review	PCa	NA	NA
[65]	Hui, F. et al.	The α1-adrenergic receptor antagonist Doxazosin inhibits EGFR and NF-κB signalling to induce breast cancer cell apoptosis	In vitro	Breast cancer	Doxazosin	Doxazosin reduces phosphorylation of EGFR and decreases pERK1/2 levels, NF-κB, AP-1, SRE, E2F and CRE-mediated transcriptional activity. Doxazosin also decreased phosphorylated retinoblastoma (pRb) protein expression, providing a potential mechanism for the doxazosin-mediated G0/G1 cell cycle arrest. Quinazoline ring structure is similar to the EGFR tyrosine kinase inhibitors. Doxazosin appears to be safe in normal cells due to the main target being EGFR and NF-κB signalling which has greater activation in cancer cells.
[66]	Park, M. et al.	The antihypertension drug Doxazosin suppresses JAK/STATs phosphorylation and enhances the effects of IFN-α/γ-induced apoptosis	In vitro, In vivo (mice)	Ovarian cancer	Doxazosin	Doxazosin significantly suppressed tumour growth in an ovarian cancer cell xenograft mouse model (50%–65% reduction in tumour size), inducing apoptotic cell death by up-regulating the expression of p53. There was no additional liver toxicity or loss of body weight. In vitro identified JAK/STAT signaling as potential mediators underlying the anti-tumour effect of doxazosin.
[67]	Kawahara, T. et al.	Silodosin inhibits prostate cancer cell growth via ELK1 inactivation and enhances the cytotoxic activity of gemcitabine	In vitro	Prostate Cancer	Silodosin and gemcitabine	Silodosin treatment reduced the expression/activity of ELK1 in these cells as well as viability of AR-positive cells, but not the viability of AR-negative or ELK1 negative cells. Interestingly silodosin significantly increased the sensitivity to gemcitabine, but not cisplatin or docetaxel. ELK1 is likely activated in prostate cancer cells and promote tumour progression. Furthermore, silodosin that inactivates ELK1 in prostate cancer cells not only inhibits their growth but also enhances the cytotoxic activity of gemcitabine. Thus, ELK1 inhibition has the potential of being a therapeutic approach or prostate cancer.
[68]	Kawahara, T. et al.	Silodosin inhibits the growth of bladder cancer cells and enhances the cytotoxic activity of cisplatin via ELK1 inactivation	In vitro	Bladder Cancer (ELK-1 positive urothelial carcinoma)	Silodosin + cisplatin	Involvement of ELK1 in bladder cancer progression via modulation cell proliferation/apoptosis, migration and invasion. In bladder and prostate cancers, ELK1 was shown to induce the proliferation of cells only with an activated androgen receptor). Silodosin was found to not only inhibit cell viability and migration, but also enhance the cytotoxic activity of cisplatin in bladder cancer lines via inactivating ELK1. The results suggest that combined treatment with silodosin is useful for overcoming chemoresistance in patients with ELK-1 positive urothelial carcinoma receiving cisplatin.
[69]	Iwamoto, Y. et al.	Oral naftopidil suppresses human renal-cell carcinoma by inducing G(1) cell-cycle arrest in tumor and vascular endothelial cells	In vitro, in vivo (mice)	Renal Cell Carcinoma (ACHN, Caki-2) ACHN	Naftopidil	Naftopidil, but not tamsulosin, was found to inhibit proliferation of renal cancer cells via induction G1 cell cycle arrest in in vitro studies.
[70]	Sakamoto, S. et al.	Anoikis disruption of focal adhesion-Akt signaling impairs renal cell carcinoma	In vitro	Renal cancer 786-0, Caki	Doxazosin and derivatives	Quinazoline-based drugs trigger anoikis in renal cancer cells by targeting the focal adhesion survival signalling.
[71]	Takara, K. et al.	Effects of α-adrenoceptor antagonist Doxazosin on MDR1-mediated multidrug resistance and transcellular transport	In vitro	Human cervical carcinoma (HeLa, Hvr100-6)	Doxazosin, prazosin, terazosin	Co-treatment of chemotheraputics (vinblastine and paclitaxel) with doxazosin (1 µM) enhanced chemosensitivity of overexpressing multi-drug resistant HeLa cells, Hvr100-6. On the other hand, prazosin (1 µM) was found to partially reverse cells sensitivity to vinblastine when used in combination, by dose-dependently increasing intracellular accumulation of chemotheraputics. Whereas terazosin had no effect. All other combinations of chemotheraputic and α1-antagonists were found to have little or no effect on chemosensitivity. Over all this study suggests that doxazosin thus may partly reverse drug resistance by inhibiting MDR-1-mediated drug efflux, and in turn, contribute to maintenance of intracellular cytotoxic concentrations.
[72]	Powe, D. et al.	α- And β-adrenergic receptor (AR) protein expression is associated with poor clinical outcome in breast cancer: an immunohistochemical study	In vitro	Breast cancer	α-Antagonists	α Antagonists were found to inhibit proliferation and induce apoptosis in vitro.
[73]	El Sharkawi, F. et al.	Possible anticancer activity of rosuvastatine, Doxazosin, repaglinide and oxcarbazepin	In vitro	MCF7, HeLa, HepG2,EACC	Doxazosin	Doxazosin was most effective in the EACC line exhibiting 100% inhibition of cell proliferation. Specific mechanisms of action are not reported or discussed.
[74]	Kanno, T. et al.	1-[2-(2-Methoxyphenylamino) ethylamino]-3-(naphthalene-1-yloxy)propan-2-ol as a potential anticancer drug	In vitro	Bladder, prostate, MPM, lung, hepatoma, gastric, renal and colorectal cancer cell lines Caco-2 and CW2	Naftopidil	This study is discussed in review above. Discuses caspase activation and cell death.
[75]	Kaku, Y. et al.	The newly synthesized anticancer drug HUHS1015 is useful for treatment of human gastric cancer	In vitro, in vivo (mice)	Gastric cancer (MKN45 and MKN28)	HUHS1015 (naftopidil analogue)	HUHS1015 treatment caused upregulation of TNFα receptor and apoptosis was observed in both MKN28 and MKN45. However, no caspase activation was observed in MKN28, indicating that HUHS1015 resulted in caspase-dependent and independent apoptosis activity. Mice bearing MKN45 tumours had higher survival rates when treated with HUHS1015 compared to those treated with cisplatin, paclitaxel and irinotecan.
[76]	Kaku, Y. et al.	HUHS1015 Suppresses Colonic Cancer Growth by Inducing Necrosis and Apoptosis in Association with Mitochondrial Damage	In vitro, in vivo (mice)	Colon cancer (Caco-2, CW2 cells)	HUHS1015 (naftopidil analogue)	HUHS1015 triggered apoptosis in colon cancer Caco-2 and CW2 cells by disrupting the mitochondrial membrane potential, lowering ATP levels, cytochrome c release, and initiation of the caspase cascade. In addition, HUHS1015 increased the number of cells in sub-G_1_ phase of cell cycling, which corresponded to apoptosis in both cell lines. In vivo mice studies demonstrated that treatment with HUHS1015, but not naftopidil, delayed colonic tumour growth compared to untreated controls. Furthermore, the authors report 100% survival rate for mice with colonic xenograft tumours treated with HUHS1015 or naftopidil, which was higher than control (89% survival).
[77]	Shen, S. et al.	Effects of α-adrenoreceptor antagonists on apoptosis and proliferation of pancreatic cancer cells in vitro	In vitro	Pancreatic cancer (PC-2 and PC-3)	Yohimbine and urapidil (α1- and α2-adrenoreceptor antagonists)	Yohimbine induced apoptotic cytotoxicity of both pancreatic PC-3 and PC-3 pancreatic cancer. In contrast, urapidil was only cytotoxic to PC-2 cells. However, the positive control 5-FU, was more cytotoxic than yohimbine in the conditions tested.
[78]	Masachika, E. et al.	Naftopidil induces apoptosis in malignant mesothelioma cell lines independently of α1-adrenoceptor blocking	In vitro	Meso-thelial cancer	Naftopidil, prazosin	Naftopidil and prazosin both have the potential to induce apoptosis via activating caspase-3 and caspase-8, but not caspase-9, independent of α1 blocking activity in mesothelioma cells.
[79]	Fuchs, R. et al.	The cytotoxicity of the α1-adrenoceptor antagonist prazosin is linked to an endocytotic mechanism equivalent to transport-P	In vitro	K562 cells erythroleukemia, LNCaP (PCa)	Prazosin/QAPB (fluorescent analogue of prazosin)	Prazosin has been shown to be a substrate for an amine uptake mechanism called transport-P. The fluorescent analogue of prazosin, QAPB was associated with endocytic mechanism of prazosin/QAPB similar transport-P. Prazosin/QAPB was able to induce caspase 8 activation (apoptosis) and tabulation of lysosomes in LNCaP cells. The cytotoxic actions of prazosin was inhibited by chloroquine (a lysomototropic drug) and bafilomycin (transport-P inhibitor). This indicates that transport-p-mediated uptake, and subsequent endosome/lysosome accumulation and caspase activation underlies prazosin-induced LNCaP and/or K562 toxicity.
[80]	Albinana, V. et al.	Propranolol reduces viability and induces apoptosis in hemanglioblastoma cells from von Hippel-Lindau patients	In vitro	Hemanglio-blastoma, cervical cancer HeLa9XHRE	Propranolol (β-blocker)	Propranolol treatment resulted in cytotoxicity and caspase-mediated apoptosis (50–100 µM, 48 h treatment) of hypoxia response element-transfected HeLa 9XHRE cells. Similar findings were also observed in hemanglioblastoma cells. Overall, the authors suggests these effects may due in part to the inhibitory effect of HIF1 transcription and protein expression in HeLA9XHRE and hemanglioblastoma cells.
[81]	Staudacher, I. et al.	HERG K+ channel-dependent apoptosis and cell cycle arrest in human glioblastoma cells	In vitro	Glioblastoma (LNT-229, U87MG)	Doxazosin, terazosin	Doxazosin was found to induce apoptosis and G0/G1 cell cycle arrest of glioblastoma LNT-229 and U87MG cells in a time and concentration dependent manner. Also, blocking of doxazosin binding to hERG by the non-apoptotic hERG ligand, terazosin, rescued glioblastoma cells from doxazosin-induced apoptosis. The apoptotic effect of doxazosin was marked by the activation of pro-apoptotic factors/signalling (phospho-erythropoietin-producing human hepatocellular carcinoma receptor tyrosine kinase A2, phospho-p38 mitogen-activated protein kinase, growth arrest and DNA damage inducible gene 153, cleaved caspases 9, 7, and 3), and by inactivation of anti- apoptotic poly-ADP-ribose-polymerase, respectively. Overall, this study suggests doxazosin is a hERG antagonist, which results in the activation of apoptotic signaling cascade.
[82]	Fuchs, R. et al.	The anti-hypertensive drug prazosin induces apoptosis in the medullary thyroid carcinoma cell line TT	In vitro	Medullary thyroid carcinoma	Prazosin	Prazosin (24 h, ≥15 µM) was found to induce caspase-3/7 activation and apoptosis of medullary thyroid carcinoma cells (α1A and α1B adrenoceptors-positive). This cytotoxicity was associated with morphological changes such as long polar needle-shaped polar protrusion fibers, an increased in number of intracellular vacuoles and detachment. The fibres present in treated cells seem to impair mobility of the cell and were associated with prazosin-mediated caspase activation. Prazosin was also found to have a similar morphological effect on normal human fibroblasts, suggesting a lack of specificity and risk of cytotoxicity to non-cancerous cells.
[83]	Tahmatzopoulos, A. et al.	Effect of terazosin on tissue vascularity and apoptosis in transitional cell carcinoma of bladder	Observational Cohort	Transitional cell carcinoma (TCC) of the bladder	Terazosin	Pathological specimens of 24 men who underwent radical cystectomy for transitional cell carcinoma of the bladder were evaluated for terazosin-induced anti-cancer effects. For this study, patients with a history of 5a-reductase inhibitor use were excluded. For men who were never exposed to terazosin (15 men), markers of apoptosis were limited in the tumour specimens of these men. In contrast, terazosin exposure prior to cystectomy (9 men, 2–10 mg/day; 3–60 months) was associated with a statistically significant increase in tumour apoptosis. Terazosin treatment also significantly decreased microvascular density (MVD) in approximately 27% of specimens compared to specimens of unexposed men.
N/A	Bajek, A. et al. (2011)	Prostate epithelial stem cells are resistant to apoptosis after α1-antagonist treatment. The impact for BPH patients	In vitro	Prostate cancer	Doxazosin	Doxazosin induced apoptosis in co-cultures of progenitor (type of stem cell) and differentiated epithelial cells. However, progenitor cells were not susceptible to apoptosis, which can be a reason of treatment failure in BPH patients.
N/A	Minarini, A. et al. (2006)	Recent advances in the design and synthesis of prazosin derivatives		-	-	Found to be irrelevant to our research but still of interest.

## Supplementary Materials

Supplementary materials can be found at www.mdpi.com/1422-0067/17/8/1339/s1.

## Appendix A

**Table A1:** Inclusion/exclusion search terms/filters included in methodology.

Search Term 1: Agent	Search Term 2: Target Tissue	Search Term 3: Action
Alfuzosin α Adrenergic antagonist α Adrenoreceptor blockers α Blocker Doxazosin Naftopidil Phentolamine Prazosin Silodosin Terazosin	Adenocarcinoma Cancer Carcinoma Neoplasm Prostate cancer	Anoikis Anti-angiogenic Anti-proliferative Anticancer Antineoplastic Apoptosis Cytotoxic
Filters Applied in PubMed		
Text—Full text Publication Date—2000–2016 Language—English Subjects—Cancer Search Fields—Title/Abstract		
Exclusion terms: In Title/Abstract		
AG1478, α-methyl-DL-tryptophan, α-linolenic, α-methyltryptophan, Biscoumarins, BYL719, Calcium channels, Cardiotoxic, Chitosan, Gambogic acid, Glyceollin, Hepatocarcinogens, HhAntag691, Insulin-like growth factor 1 receptor, LSC, Mast cells, PSC833, R482G isoform, Raloxifene, Stapling, Stilbenes, Toremifene, Triphosphate-binding		
Combined Search Terms		
(α adrenergic antagonist OR α adrenoreceptor blocker OR α blocker OR Prazosin OR Doxazosin OR Naftopidil OR Phentolamine OR Alfuzosin OR Terazosin OR Silodosin) AND (Neoplasm OR Cancer OR Prostate cancer OR Carcinoma OR Adenocarcinoma) AND (Anoikis OR Antineoplastic OR Apoptosis OR Anticancer OR Anti-angiogenic OR Anti-proliferative OR Cytotoxic) NOT (Calcium channels OR Cardiotoxic OR LSC OR Biscoumarins OR Glyceollin OR Chitosan OR toremifene OR triphosphate-binding OR α-linolenic OR α-methyl-DL-tryptophan OR HhAntag691 OR α-methyltryptophan OR raloxifene OR AG1478 OR R482G isoform OR PSC833 OR Mast cells OR Gambogic acid OR hepatocarcinogens OR insulin-like growth factor 1 receptor OR stapling OR BYL719 OR stilbenes)		
