# Supplementary material for: The Role of α1-Adrenoceptor Antagonists in the Treatment of Prostate and Other Cancers
Source: Int J Mol Sci. 2016 Aug 16;17(8):1339. doi: 10.3390/ijms17081339 (PMC5000736; doi:10.3390/ijms17081339)
Supplement: Supplementary file 1 [file ijms-17-01339-s001.pdf]

Supplementary Materials: The Role of α1-Adrenoceptor Antagonists in the Treatment of Prostate and Other Cancers

Mallory Batty, Rachel Pugh, Ilampirai Rathinam, Joshua Simmonds,Edwin Walker, Amanda Forbes, Shailendra Anoopkumar-Dukie, Catherine M. McDermott, Briohny Spencer, David Christie and Russ Chess-Williams

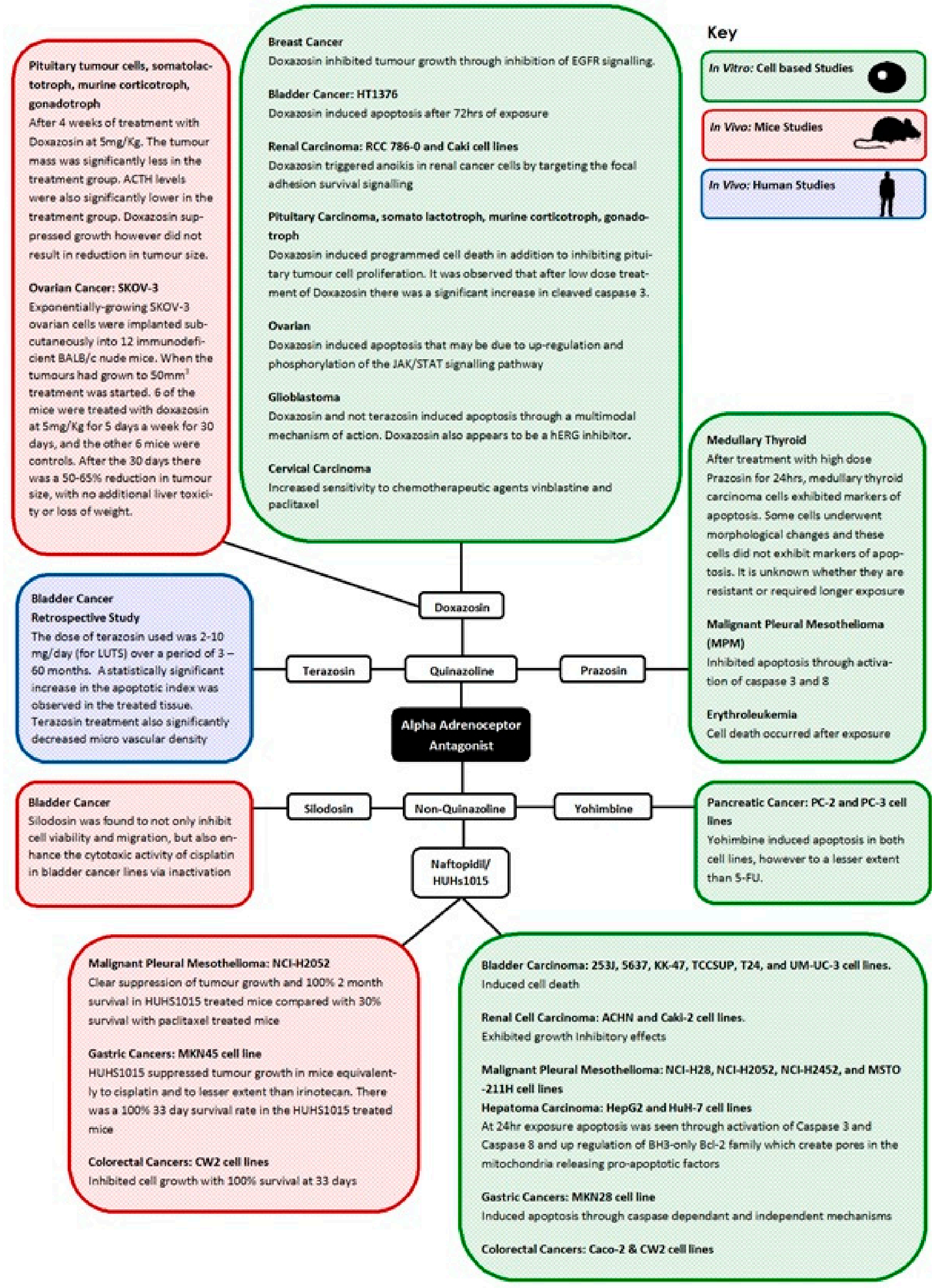

Figure S1. Evidence for cytotoxic/anticancer effects of α-adrenoreceptor antagonists in cancers other than prostate cancer.
